# Ensuring image integrity in the digital age

**DOI:** 10.1002/2211-5463.13495

**Published:** 2022-12-01

**Authors:** Duncan E. Wright, Miguel A. De la Rosa

**Affiliations:** ^1^ FEBS Open Bio Editorial Office Cambridge UK; ^2^ Institute for Chemical Research (IIQ), Scientific Research Centre Isla de la Cartuja (cicCartuja) Universidad de Sevilla‐CSIC Spain

## Abstract

*FEBS Open Bio* and our fellow FEBS Press journals have a strong commitment to maintaining the integrity of the scientific literature. The life sciences, in particular, are suffering from an ongoing reproducibility crisis, and this may in part be fuelled by mistakes, manipulation or outright fabrication of the presented data. We were recently made aware of several articles published in *FEBS Open Bio* that appear to contain full or partial duplications of images from other published articles in a different scientific context. In most of these cases, the duplications were taken from previously published papers. After thorough investigation and subsequent discussion within FEBS Press and with Wiley's Integrity in Publishing Group, we have retracted most of these articles.

## Graphical abstract

*FEBS Open Bio* has a strong commitment to maintaining the integrity of the scientific literature. We were recently made aware of several articles published in *FEBS Open Bio* that appear to contain full or partial duplications of images from other published articles in a different scientific context. After thorough investigation, we have retracted most of these articles.

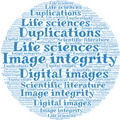

*FEBS Open Bio* and our fellow FEBS Press journals are strongly committed to maintaining the integrity of the scientific literature. The life sciences, in particular, are suffering from an ongoing reproducibility crisis [1], and this may in part be fuelled by mistakes, manipulation or outright fabrication of the presented data. In an ideal world, any figure anomalies would be detected during the peer‐review process, but the ongoing increase in retraction of life science articles [2], an alarming number of which are retracted for fraud [3], indicates that many issues with images are not detected until after publication.

Fraud and honest errors have always posed a threat to the integrity of the scientific literature, but these dangers have grown considerably in the digital age. While unscrupulous authors once had to craft a manipulated figure painstakingly by hand, they now merely require a rudimentary understanding of Photoshop to create fake images that may deceive the untrained eye. Equally, the ease at which digital images can be acquired may result in an image glut that facilitates mistakes. One particularly insidious threat to the integrity of the scientific literature is the emergence of paper mills, organisations that create fake manuscripts and sell them to researchers in need of a quick publication [4].

In 2017, Jana Christopher joined FEBS Press as Data Integrity analyst for the four journals, including *FEBS Open Bio*. She routinely screens the figures of accepted manuscripts before publication for potential issues and also inspects published articles when concerns have been raised by a third party. Jana's expertise and diligence have resulted in the identification of hundreds of figure anomalies, both honest errors and outright manipulations. Her efforts go largely unnoticed, as manuscripts identified to have figure problems are either corrected before publication (in the event of honest mistakes), or manuscripts may be rejected outright when manipulation is evident, or a compelling explanation and/or raw data are lacking.

While it may be possible to detect duplications *within* a paper, duplications of images *between* papers are substantially trickier to spot: one would not only need to have seen the original image previously (a drop in the ocean when we consider the vast number of multi‐figure research articles published each year), but also be in possession of a truly phenomenal memory! While the use of plagiarism detection software to compare the text of submitted manuscripts to the published literature is now common practice amongst many publishers (including FEBS Press), the substantially greater processing power required to detect image duplications between papers had rendered this little more than a pipedream until recently. The ongoing development of AI‐based software to detect image duplications is now beginning to help journals and independent investigators detect previously unsuspected problems in the literature. The work of initiatives such as the STM Integrity Hub will also be pivotal to tackling these issues and preventing them from being passed from journal to journal (https://www.stm‐assoc.org/stm‐integrity‐hub/).

We were recently made aware of articles published in *FEBS Open Bio* that contain full or partial duplications of images from other published articles by different authors *in a different scientific context*. After thorough investigation in accordance with COPE Guidelines (https://publicationethics.org/resources/flowcharts/image‐manipulation‐published‐article) involving the authors and their institutions, and subsequent discussion within FEBS Press and with Wiley's Integrity in Publishing Group, we have retracted most of these articles. The decision to retract is never taken lightly, but was unambiguous in these cases.

The Editorial Office of *FEBS Open Bio* will continue to carefully screen newly submitted manuscripts for potential problems and investigate concerns with figures in published articles. We hope that the research community will support both us and other journals in our efforts by learning about the tactics used by paper mills, ensuring best practice in their own research, and maintaining a critical eye when reviewing manuscripts. Science may well be self‐correcting, but it can only do this through a collective effort by researchers, publishers and institutions.

## References

[1] Baker M . 1,500 Scientists lift the lid on reproducibility. Nature. 2016;533(7604):452–4. 10.1038/533452a 27225100

[2] Yeo‐Teh NSL , Tang BL . Sustained rise in retractions in the life sciences literature during the pandemic years 2020 and 2021. Publications. 2022;10(3):29. 10.3390/publications10030029

[3] Fang FC , Steen RG , Casadevall A . Misconduct accounts for the majority of retracted scientific publications. Proc Natl Acad Sci USA. 2012;109(42):17028–33. 10.1073/pnas.1212247109 23027971 PMC3479492

[4] Christopher J . The raw truth about paper mills. FEBS Lett. 2021;595(13):1751–7. 10.1002/1873-3468.14143 34180058

